# The shallow structure of Mars at the InSight landing site from inversion of ambient vibrations

**DOI:** 10.1038/s41467-021-26957-7

**Published:** 2021-11-23

**Authors:** M. Hobiger, M. Hallo, C. Schmelzbach, S. C. Stähler, D. Fäh, D. Giardini, M. Golombek, J. Clinton, N. Dahmen, G. Zenhäusern, B. Knapmeyer-Endrun, S. Carrasco, C. Charalambous, K. Hurst, S. Kedar, W. B. Banerdt

**Affiliations:** 1 Swiss Seismological Service (SED), ETH Zurich, Zurich, Switzerland; 2 Institute of Geophysics, ETH Zurich, Zurich, Switzerland; 3 Jet Propulsion Laboratory, California Institute of Technology, Pasadena, CA 91109 USA; 4 Bensberg Observatory, University of Cologne, Bergisch Gladbach, Germany; 5 Department of Electrical and Electronic Engineering, Imperial College London, London, UK; 6 Present Address: Federal Institute for Geosciences and Natural Resources (BGR), Hanover, Germany

**Keywords:** Inner planets, Seismology, Seismology

## Abstract

Orbital and surface observations can shed light on the internal structure of Mars. NASA’s InSight mission allows mapping the shallow subsurface of Elysium Planitia using seismic data. In this work, we apply a classical seismological technique of inverting Rayleigh wave ellipticity curves extracted from ambient seismic vibrations to resolve, for the first time on Mars, the shallow subsurface to around 200 m depth. While our seismic velocity model is largely consistent with the expected layered subsurface consisting of a thin regolith layer above stacks of lava flows, we find a seismic low-velocity zone at about 30 to 75 m depth that we interpret as a sedimentary layer sandwiched somewhere within the underlying Hesperian and Amazonian aged basalt layers. A prominent amplitude peak observed in the seismic data at 2.4 Hz is interpreted as an Airy phase related to surface wave energy trapped in this local low-velocity channel.

## Abstract (web summary)

We invert Rayleigh wave ellipticity curves extracted from ambient seismic vibrations at the InSight landing site to resolve, for the first time on Mars, the shallow subsurface to around 200 m depth. While our seismic velocity model is largely consistent with the expected stacks of lava flows, we find a seismic low velocity zone at about 30 to 75 m depth that we interpret as a sedimentary layer sandwiched between layers of basalt flows.

## Introduction

Mars has been the target of a large number of planetary science missions involving flybys, orbiters, landers, and rovers that have focused on surface and atmospheric remote sensing as well as surface geochemistry and mineralogy. NASA’s InSight (Interior Exploration using Seismic Investigations, Geodesy and Heat Transport) mission is the first to specifically target the subsurface using seismic methods^1^ (see Supplementary Fig. 1 for a map of the landing region in Elysium Planitia), deploying a very broad-band seismometer^2^ (SEIS). SEIS operates continuously with the primary goal to detect marsquakes in order to quantify Martian seismicity^3,4^ and to infer the interior structure of Mars at all scales^5^. First results from the analysis of the SEIS data provide new information on the large-scale internal structure, physical properties, and tectonic activity of Mars^3,6^. Seismic studies of the shallow subsurface around the InSight landing site so far^5^ have been limited to the uppermost 10−20 m using seismic-traveltime measurements^7^ and ground compliance estimates^8,9^, leaving structures at few tens to several hundreds of meters depth uncharted.

Detailed near-surface models can provide direct constraints for understanding the processes that formed Elysium Planitia. Such models are required to understand the stratigraphy and the role of volcanism as well as sedimentation in the transition zone of the dichotomy between ancient southern heavily cratered highlands and low-standing younger, smoother northern plains. The surface plains near the dichotomy boundary on which InSight landed is mapped as an Early Hesperian Transition (3.7−3.4 Ga) unit that could be volcanic or sedimentary deposits^10^ from the 2 km high dichotomy to the south^11^. Geologic mapping in high-resolution images, rocky ejecta craters, mafic minerals in visible and infrared spectra and the presence of wrinkle ridges all argue that the plains around InSight are underlain by about 200−300 m of layered basalts^12^. Furthermore, small craters without rocky ejecta, images of nearby escarpments and thermophysical properties argue for about 3 m of overlying dominantly sandy, impact-generated regolith^13^. With its geophysical instrument suite, InSight is the first mission capable of investigating the near-surface beyond a few centimeters of depth. Such information will provide valuable ground truth to orbital-data based surface and subsurface models.

The ground at the InSight landing site is in continuous motion, even during periods without marsquake-related shaking. The composition of the Martian ambient seismic wavefield is different from the terrestrial case, as two of the main sources of seismic ambient vibrations on Earth, oceans and anthropogenic activity, are absent. The oceans on Earth act as a very efficient way to transfer atmospheric energy into seismic energy. On Mars, wind−surface interaction is the main source of seismic ambient vibrations^14^. InSight observations show that the level of ambient seismic vibrations on Mars is low due to the absence of oceans, the hundred-fold thinner atmosphere and the around 50% reduction in the solar irradiation compared to Earth. The minimum seismic noise level is found between 0.05 and 5 Hz^5^, with amplitudes significantly below the Earth low noise model^15^.

SEIS records ambient vibrations, including those generated by lander motions, that create a broad-band background signal. The amplitude of the low-frequency (0.03−1 Hz) ambient seismic vibrations recorded by SEIS has been observed to be frequency-dependent and strongly related to environmental effects such as wind during the day^16^. In contrast, in the evening, the observed winds often drop significantly and during this period and for the studied low-frequency window between 0.3 and 1 Hz, it was concluded^16^ that the recorded polarized seismic signals may indeed correspond to the wavefield of the Martian ambient seismic background vibrations.

**Fig. 1:**
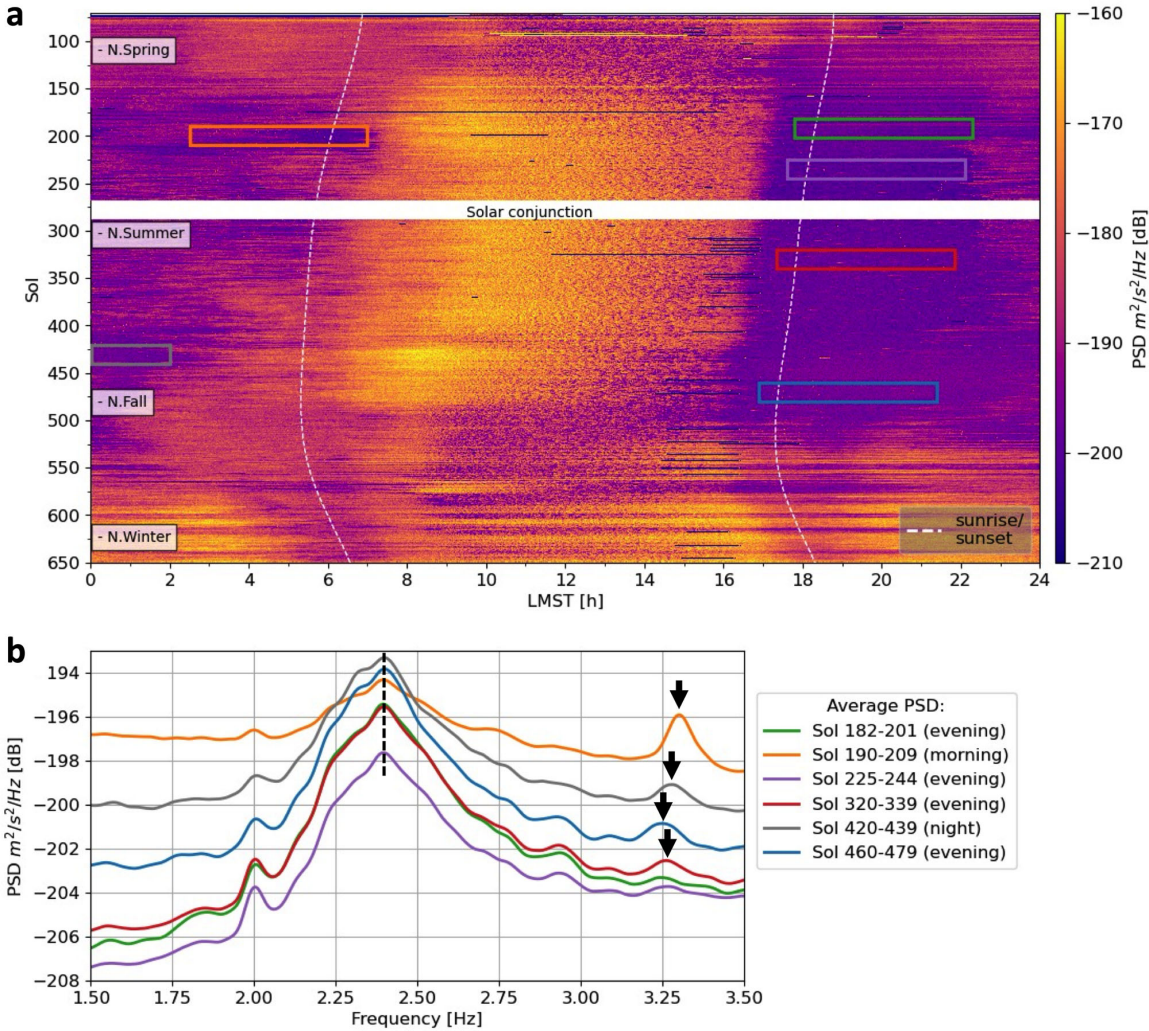
2.4 Hz mode observation across the InSight mission. **a** Vertical-component energy between 2.3 and 2.5 Hz for sols 80−650. Each row corresponds to the data from one sol plotted against local mean solar time (LMST). Vertical white dashed lines indicate sunrise and sunset. **b** Power spectral density (PSD) plots averaged over 20 sols extracted for the time windows marked in the spectrogram in (**a**). Note specifically the stable shape of the peak around 2.4 Hz across the entire mission duration. Evenings are generally quieter and more stable than any other time of day. The peak power level of the 2.4 Hz mode marked by the dashed line weakly depends on the wind speed^17^. In comparison, the lander-related mode at 3.3 Hz (black arrows) changes its peak frequency and amplitude level considerably depending on wind and temperature.

**Fig. 2:**
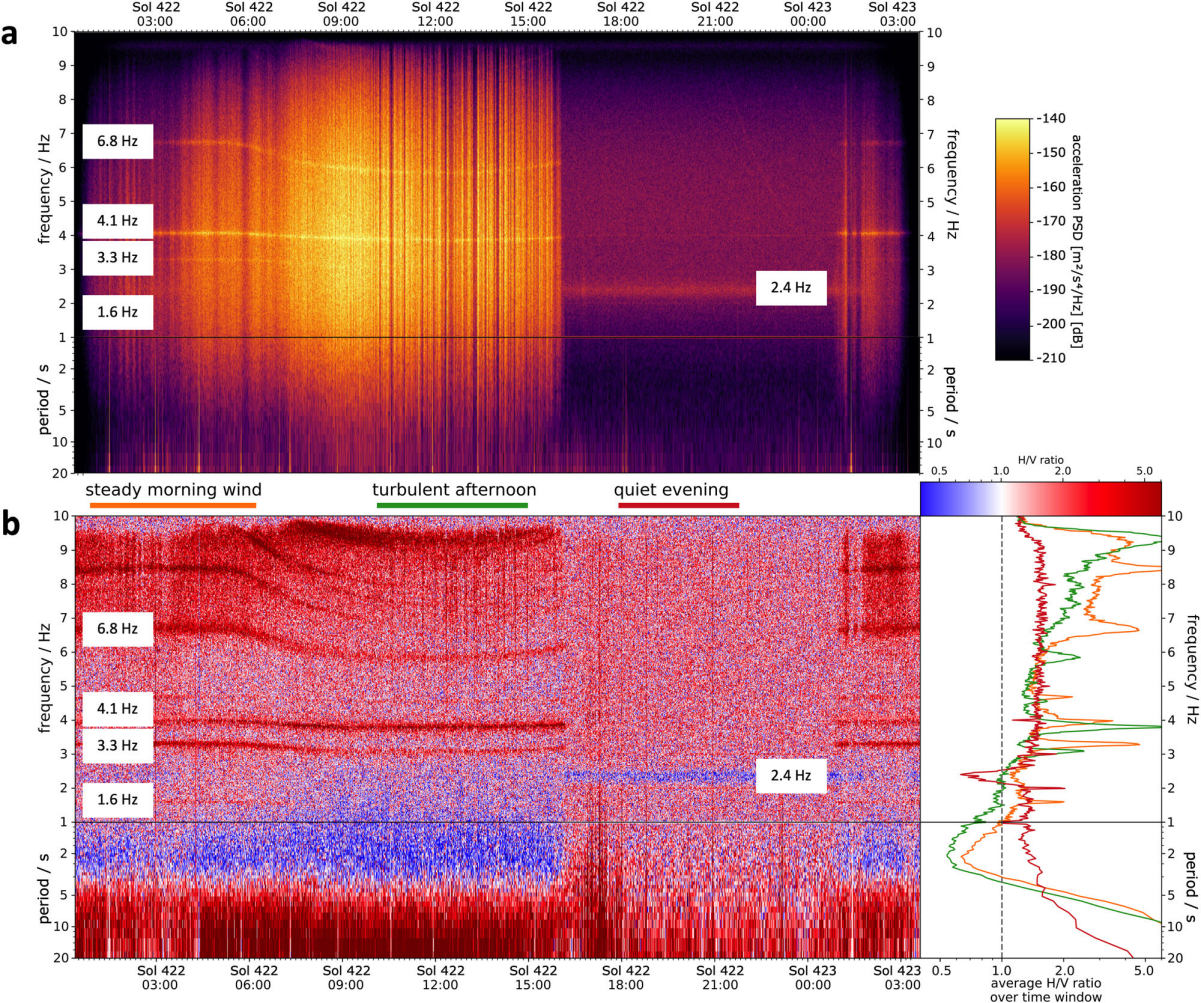
Frequency-domain characteristics of sols 422 and 423 SEIS data. **a** Vertical-component spectrogram of sols 422 and 423. Typically, mornings and afternoons show high levels of wind-induced noise, while evenings and nights are characterized by significantly lower ambient noise levels due to largely absent local winds. Note the lander-related modes (e.g., at 1.6, 3.3, 4.1, 6.8 Hz) that show a time-dependent change of their resonance frequencies. The lander-related modes correlate with the wind activity, suggesting that winds shaking the lander are the cause of these resonances. **b** H/V ratio for the same data as in (**a**). During quiet time intervals such as in the evening hours, the ambient vibration power spectrum is relatively flat with the prominent exception of a distinct H/V trough at around 2.4 Hz. (right) Three H/V curves extracted for two windy and one quiet period of sols 422/423. The orange curve corresponds to the steady morning wind time window, the green curve to the turbulent afternoon time window and the red curve to the quiet evening time window (see colored bars marking the time windows).

Across the first Martian year of InSight operation, highly repeatable wind patterns were observed with steady winds during the morning and gusty winds every afternoon resulting in periods of increased high-frequency noise (Fig. 1a). Example spectrograms of single sols displayed in Fig. 2a show that these noisy time periods are characterized by a number of discrete spectral peaks (most prominently at 3.3, 4.1 and 6.8 Hz^17^; sol is a solar day on Mars). These peaks are interpreted as eigenmodes of mechanical lander parts and the lander’s solar arrays^17,18^ and cannot be produced by the underground structure below the station.

During summer months at the landing site (sols 180−450), extended periods with very low winds occur each evening beginning around dusk (Fig. 1a). In absence of local wind-induced noise, the ambient seismic spectrum is relatively flat between 1.5 and 8 Hz, with the prominent exception of a distinct spectral peak at 2.4 Hz in the vertical-component data (Figs. 1 and 2). This peak at 2.4 Hz is clearly distinct from the lander-related eigenmodes as its shape is much broader, it is predominantly vertically polarized in contrast to the primarily horizontally polarized lander-related modes, and, most importantly, is not temperature-modulated in frequency in contrast to the lander-related modes^17^ (Fig. 1b). Furthermore, the peak at 2.4 Hz is the only resonance phenomenon excited by marsquakes^3,4,17^. A weak proportionality is observed between the amplitude of the 2.4 Hz peak and the measured wind between 4 and 6 m/s; furthermore, the 2.4 Hz peak is unaffected by short wind bursts and strong winds^17^. The wind-induced amplitude increase of the 2.4 Hz peak is about 200−500 times weaker than the increases observed for the resonances that are recognized to originate on the lander^17^. Nevertheless, a mechanical resonator such as the solar panels, assuming a different temperature- and wind-dependent behavior than the lander-related modes, has been discussed within the SEIS team as an alternative mechanism for the 2.4 Hz peak. Still, the 2.4 Hz peak is the only observed ambient seismic vibration that could be produced as the natural seismic response expected by the heterogeneous local subsurface at the landing site in Elysium Planitia.

The Martian ambient seismic vibrations are expected to be generated mainly by the wind interacting with topography^14^, and, similar to Earth, to be composed of all types of seismic waves^19–21^. Because the source is at the surface, the ambient vibration seismic wavefield will predominantly consist of surface waves, namely Rayleigh and Love waves, modulated by the local subsurface structure on a scale of a few wavelengths around an observation point. Rayleigh waves have an elliptical motion in the vertical plane and their frequency-dependent elliptical polarization depends on the local subsurface properties^22–25^.

On Earth, the horizontal-to-vertical spectral ratio (H/V) of ambient seismic vibrations has been used for decades in engineering seismology for site characterization^20,26^. Numerical and field studies have shown that the H/V ratio is closely related to Rayleigh wave ellipticity, which is determined by the local subsurface layering^27^. Several techniques have been developed to extract the Rayleigh wave ellipticity from an ambient wavefield of unknown composition, suppressing, for example, Love wave contaminations of the horizontal components (e.g., Single-station determination of Rayleigh wave ellipticity by using the random decrement technique, RayDec^28^). Furthermore, the inversion of the ellipticity values for the subsurface structure has been a topic of intense research^29–33^. Nevertheless, Rayleigh wave ellipticity curves alone are not sufficient to retrieve the subsurface structure without additional constraints^30,34^ because ellipticity is a unitless property and, hence, does not carry information on absolute velocities of the underground, but only on the relative shape of the velocity profile. Given the success of H/V and Rayleigh wave ellipticity analyses on Earth, the potential to constrain the Martian subsurface structure using the ellipticity obtained by analyzing ambient vibrations was proposed before the mission^24,25^.

In this work, we perform a Rayleigh wave ellipticity analysis focusing on data recorded during a representative quiet-period time window. For the first time, using this classic seismological technique, we resolve the shallow subsurface stratigraphy at the InSight landing site in Elysium Planitia on Mars to around 200 m depth, and are able to infer aspects of the local geologic history in detail. While our seismic velocity model is largely consistent with the expected layered subsurface structure consisting of a thin regolith layer above stacks of lava flows, we find a seismic low-velocity zone at about 30−75 m depth that we interpret as a sedimentary layer somewhere within the Hesperian and Amazonian aged basalt layers. The prominent amplitude peak observed in the seismic data at 2.4 Hz is interpreted as an Airy phase related to surface wave energy trapped in this local low-velocity channel.

## Results

### Data selection

We carry out a Rayleigh wave ellipticity analysis focusing on data recorded during a representative quiet-period time window of 7 h length in the night from sol 422 to 423 (Sol 422 18:08:21 to Sol 423 00:57:07 local mean solar time (LMST), corresponding to 3 February 2020, 14:15:00 to 21:15:00 UTC). Whereas H/V curves computed from the windy periods show a large variability and are dominated by the lander-related modes, the H/V ratio for the quiet evening is stable over the whole mission. In these time periods, the local wind at the landing site is relatively low and too weak to generate lander-related disturbances by wind-induced shaking. Nevertheless, surface waves that compose the ambient vibration field at the sensor can still be generated by distant sources.

**Fig. 3:**
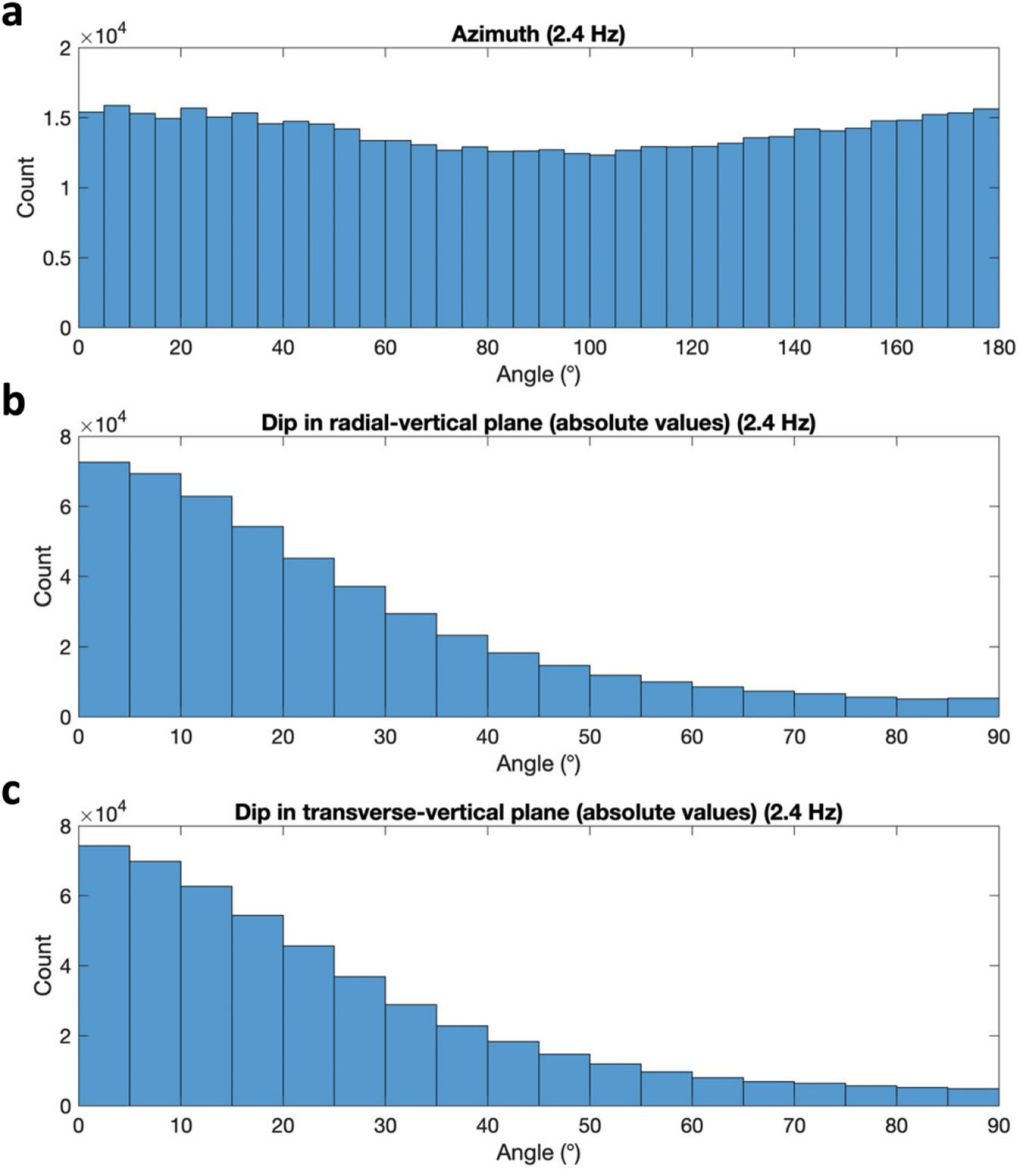
Polarization analysis of the entire data window from sol 422 to 423 at a frequency of 2.4 Hz. **a** Propagation azimuth between 0° and 180°, with a 180° ambiguity. **b** Tilt angle of the major semiaxis of the ellipse from the vertical in propagation (radial) direction. **c** Tilt angle of the major semiaxis of the ellipse from the vertical in transverse direction.

The H/V curves are largely constant for the frequency band between around 1.5 and 8 Hz except for the prominent trough at 2.4 Hz (Fig. 2b). Although a direct interpretation and inversion of H/V curves is possible^35^, this requires an isotropic wavefield and equipartitioning of the seismic energy among the different wave modes^36^. Previous InSight studies suggest an isotropic wavefield for the 0.3−1 Hz band in the quiet evening hours^16^. For the evening data window used in this study, we find at 2.4 Hz predominately elliptical motion patterns in the vertical plane with no preferred propagation direction using a polarization analysis technique based on^37,38^ (Fig. 3). These observed polarization patterns indicate that Rayleigh waves generated by randomly distributed sources are likely the dominant component of the analyzed ambient vibrations wavefield at 2.4 Hz. The extracted polarization attributes are a further evidence of the subsurface-related nature of the 2.4 Hz peak, as the lander-related resonances exhibit highly repeatable polarization azimuths. Nevertheless, estimating polarization attributes depends on the chosen approach and user-defined parameters such as the analysis window, and hence comes with some uncertainty. To extract the ellipticity of Rayleigh waves from the recorded data, we use the RayDec method^28^. RayDec is a powerful tool to retrieve the Rayleigh wave ellipticity by statistical means, even in cases when isolated Rayleigh waves are difficult to identify in the measured wavefield and the overall observed polarization pattern is complicated.

**Fig. 4:**
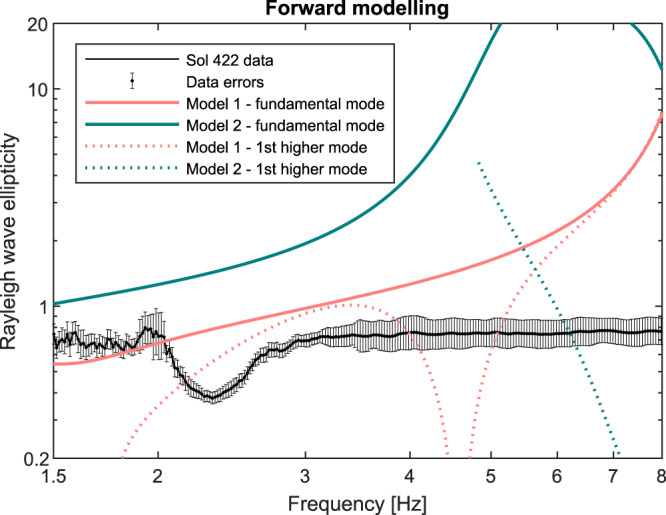
Extracted Rayleigh wave ellipticity curve compared with modeling results for pre-mission near-surface seismic models. The thick black line shows the Rayleigh wave ellipticity curve extracted for a 7-h duration time window in the evening of sol 422/423 (see Fig. 2). The vertical bars show the estimated data errors. Note that the errors were manually increased for the frequency range around 2 Hz that is known to be affected by monochromatic electronic cross-coupling noise^39^. Ellipticity curves for the fundamental and first higher Rayleigh wave modes computed for the two velocity models are shown in red and blue, respectively (models 1 and 2; Tables 1 and 2). Note that neither of the modeled Rayleigh wave ellipticity curves is able to explain the observed trough at 2.4 Hz.

The frequency-dependent Rayleigh wave ellipticity functions obtained by the RayDec method for the quiet time period are shown in Fig. 4. The shape of the curves is relatively similar to the H/V curves displayed in Fig. 2b, but the absolute ellipticity values are smaller than the H/V values. These smaller values are expected because wavefield components other than Rayleigh waves such as Love waves are mainly present on the horizontal components and are suppressed by the RayDec processing. Between 1.5 and 2.0 Hz, the curve is relatively flat with an ellipticity of about 0.7. At 2 Hz, we expect a strong influence of the first harmonics of the so-called 1-Hz tick noise, which is an electronic cross-coupling noise observed on all data acquired by SEIS^39^. Between 3 and 8 Hz, a plateau at a value of about 0.7 without significant peaks is observed.

The prominent trough at 2.4 Hz dominates the ellipticity curve between 2 and 3 Hz (Fig. 4). For comparison, on Earth, low H/V values across a wide frequency range have been reported to be related to low-velocity layers at depth^40^. For a seismic station in southern Italy, ellipticities below 1 between about 1.5 and 9.0 Hz were found^41^. The station was located on rigid conglomerates of about 15 m thickness over clays with lower velocity and a thickness of about 300 m. At another site close to Mount Etna on the island of Sicily, low H/V values were found for locations where high-velocity lava flow deposits overlay low-velocity sedimentary layers^42^.

### Forward modeling

In a first attempt to interpret the observed Rayleigh wave ellipticity curve, we tested its compatibility with layered subsurface structures derived in pre-landing studies and based on first InSight results^5,12,13,24,43–46^. In summary, the subsurface at the landing site is expected to consist of a thin (< 5 m) regolith layer on top of stacks of fractured basaltic lava flows. Below the basaltic unit, a weak sedimentary layer has been suggested at a depth of around 150−200 m based on the analysis of flooded impact craters near the lander. Loosely based on refs. ^24,45^, we established two conceptual S-wave (*v*_S_) and P-wave (*v*_P_) velocity models of the shallow subsurface (< 200 m) summarized in Tables 1 and 2 reflecting a stepwise increase in seismic velocities with depth down to the low-velocity sedimentary layer at depth (see also “Methods” section). Conceptual model 2 primarily differs from model 1 in that the seismic velocities are overall lower based on the expectation that impact cratering can be effective at cracking rock to significant depths, and that the weak and even fissile sedimentary rocks on Mars likely have low seismic velocities.

**Table 1:** Simplified near-surface reference model (referred to as model 1; loosely based on Knapmeyer-Endrun et al.^24,25,45^).

	Thickness [m]	*v*_P_ [m/s]	*v*_S_ [m/s]
Regolith	3	200	120
Blocky ejecta	10	1200	700
Fractured basalt	20	3000	1700
Intact basalt	140	5000	2850
Sediments	—	3000	1700

We computed the theoretical Rayleigh wave ellipticity curves for the two models and found that neither of these subsurface models leads to Rayleigh wave ellipticity curves that even closely resemble the observed curve with a trough at 2.4 Hz (Fig. 4). Also, the observed ellipticity curve cannot be explained by the ellipticity of the first higher Rayleigh wave mode either. These findings motivated us to perform a series of inversions of the observed Rayleigh wave ellipticity curve for the Sol 422/423 data to resolve the *v*_S_- and *v*_P_-structure of the topmost 200 m at the InSight landing site, using the reliable part of the ellipticity curve between 1.5 and 8.0 Hz. For these inversions, we assume that the extracted Rayleigh wave ellipticity curve reflects the fundamental mode only. In general, the fundamental mode of Rayleigh waves exists at all frequencies and higher modes are only present above certain frequencies, where they may be even more energetic than the fundamental mode. In that case, a mixture of different modes would be visible in the ellipticity curve as well. The flat shape of the measured ellipticity curve between 3 and 8 Hz strongly suggests that higher Rayleigh wave modes do not carry a significant amount of energy within the 3−8 Hz frequency band.

### Bayesian inversion

The inversion of the Rayleigh wave ellipticity curve to retrieve the underground 1D seismic velocity structure is a non-linear inverse problem characterized by significant inherent non-uniqueness. This inversion is particularly challenging due to ambiguous solutions, and in particular, if only very limited near-surface information is available to constrain the inversion. We perform multiple inversions of the ellipticity data in a Bayesian framework utilizing both flat and depth-dependent constrained prior expectations of the underground structure. The applied inversion technique^32^ relies on a trans-dimensional formulation of the parameter space where the number of layers is an unknown parameter itself^47,48^. The number of layers is treated as inversion parameter governed by the law of parsimony^49^; we strive for the simplest possible layered models, but not simpler than required by the data.

**Fig. 5:**
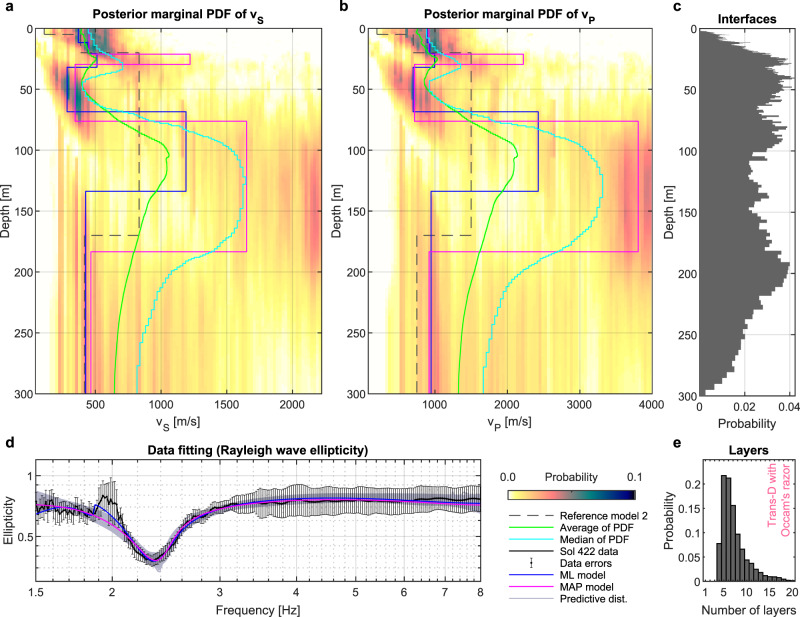
Result of the inversion of the Rayleigh wave ellipticity curve using a flat prior probability density function (PDF). **a**, **b** show the posterior marginal probability density functions of *v*_S_ and *v*_P_, respectively. **c** Histogram of the occurrence of layer interfaces. **d** Extracted (black line) and modeled ellipticity curves for models drawn from the posterior PDF (ML maximum likelihood, MAP maximum a posteriori). Vertical bars indicate the expected data error (used as inverse data weight). **e** Posterior histogram of the number of layers.

**Fig. 6:**
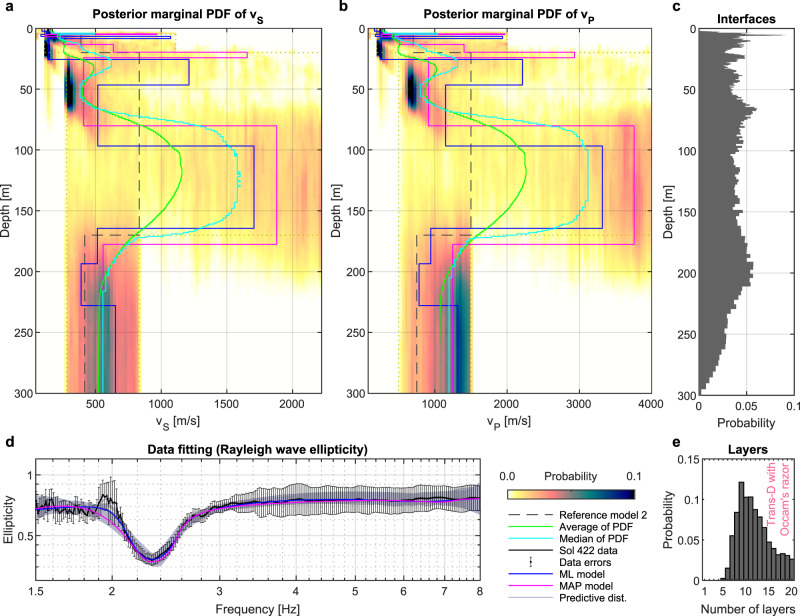
Result of the Rayleigh wave ellipticity inversion constrained by bounds from model 2. **a**, **b** show the posterior marginal probability density functions (PDF) of *v*_S_ and *v*_P_, respectively. **c** Histogram of the occurrence of layer interfaces. **d** Extracted (black line) and modeled ellipticity curves (ML maximum likelihood, MAP maximum a posteriori). Vertical bars indicate the expected data error. **e** Posterior histogram of the number of layers.

A first inversion with flat priors was used to infer the underground structure without subjective influence on the solution (Fig. 5). Additional inversion runs using parameter value constraints by depth-dependent bounds based on subsurface model 2 (Table 2; depth-dependent bounds are prescribed to parameters *v*_S_, *v*_P_ and Poisson’s ratio) and additional inversion runs with fixed numbers of layers were used to further explore the parameter space and support the inferred results (Fig. 6 and Supplementary Figs. 3 and 4). These inversion tests and results are described in detail in the “Methods” section.

**Table 2:** Modified near-surface reference model (referred to as model 2). The numbers in brackets mark the full range explored in the inversion (i.e. bounds of the depth-dependent multizonal prior PDF).

	Thickness [m]	*v*_P_ [m/s]	*v*_S_ [m/s]
Regolith	5 (3−6)	200 (80−300)	111 (40−170)
Blocky ejecta	15 (5−25)	700 (200−2000)	389 (110−1100)
Basalt	150 (100−200)	1500 (500−4000)	833 (280−2220)
Sediments	—	750 (500−1500)	417 (280−840)

### Inferred seismic velocity models

The Rayleigh wave ellipticity data depend mainly on the S-wave velocity structure below the measurement site. Hence, the inference of the *v*_S_-profile is of primary interest. The *v*_P_-profiles are constrained by plausible value ranges for the Poisson’s ratio for the expected rock and soil (i.e., 0.2−0.4). The ellipticity data reflect velocity contrasts in depth rather than absolute values of velocities, and the inversion results may be affected by trade-offs between absolute values of velocity and interface depth. Hence, a large family of different models can explain the data equally well. Furthermore, our layer-based Bayesian inversion favors simple models (i.e., with a minimum number of layers) over models with a large number of layers (i.e., a staircase-like representation of a gradual velocity change).

We focus our interpretation on common and robust features found in all inversion results displayed in Figs. 5 and 6, rather than interpreting a single final model. For illustration purposes, we display models with the least data misfit (ML model) and the maximum a posteriori (MAP) model estimate. Our MAP model estimate corresponds to the layered *v*_S_-model from the ensemble of solutions that has the smallest L1-norm misfit with the most probable posterior *v*_S_-profile^32^. The green and light blue curves (mean and median profiles, respectively) displayed in Figs. 5 and 6 provide in addition an overall impression of the probable velocity changes with depth.

Due to the limited frequency bandwidth of the ellipticity curve ranging from 1.5 to 8 Hz and lacking higher-frequency information, the shallowest part of the model is only poorly resolved. According to the guidelines of the InterPACIFIC project^50^, a surface wave inversion can constrain the velocity structure below about half of the minimum used wavelength. According to Supplementary Fig. 5, the Rayleigh wave phase velocity of the MAP model at 8 Hz is around 200 m/s, corresponding to a wavelength of about 25 m. This rough estimation indicates that we cannot constrain the shallowest 12.5 m and we are cautious not to overinterpret our results for the uppermost 20 m.

A particular feature of the extracted ellipticity curve are unusually low values below 1 (see Fig. 4), which have been found to be indicative of low-velocity layer(s). Indeed, a low-velocity layer between around 30 and 75 m depth, not present in the pre-landing models (see dashed line in Figs. 5a, b and 6a, b), was found to be a robust feature of the inversion results displayed in Figs. 5 and 6 based on various tests with different input parameters and constraints. Even though the primary feature of the Rayleigh wave ellipticity data is the prominent trough at 2.4 Hz, the constant ellipticity values and absence of additional peaks and/or troughs between 1.5 and 8 Hz additionally constrain the models. To test the robustness of the results, we performed additional inversion tests with ellipticity data from the limited frequency bands of 1.5–3 Hz and 1.5–4 Hz. These resulted in velocity profiles similar to those using the full bandwidth, but with an increased overall uncertainty, especially in the shallower part (< 20 m depth). However, a low-velocity zone was found in all tests using limited frequency bands, highlighting that this feature is related to the 2.4 Hz trough.

For the following geological interpretation, we revert to the ML and MAP models extracted from the weakly constrained and model-2 constrained inversion runs that are summarized in Fig. 6a, b.

### Regolith and coarse blocky ejecta layer

While the Rayleigh wave ellipticity inversion for frequencies below 8 Hz has a limited resolution for the topmost around 20 m depth, the comparison of forward-modeled and measured data still allows us to rule out shallow subsurface features that lead to ellipticity curves inconsistent with the actual observations. Based on such forward-modeling tests, we found that the shallowest layer with *v*_S_ < 150 m/s (*v*_P_ < 300 m/s) cannot be thicker than 1−1.5 m, and a significant increase to S-wave velocities above 400 m/s (*v*_P_ > 700 m/s) below that depth is required, otherwise a high-value Rayleigh wave ellipticity peak should be visible below 8 Hz. Both the velocity values and the relatively small thickness of the top low-velocity layer are consistent with previous compliance inversions^5,9^ and the seismic-traveltime measurements^5^, suggesting *v*_S_ and *v*_P_ values of 84–152 and 136–304 m/s close to or at the surface, respectively. Furthermore, the compliance inversions indicate a relatively thin uppermost layer of less than about 2 m thickness and a structural discontinuity between 0.7 and 7 m depth^5,9^.

**Fig. 7:**
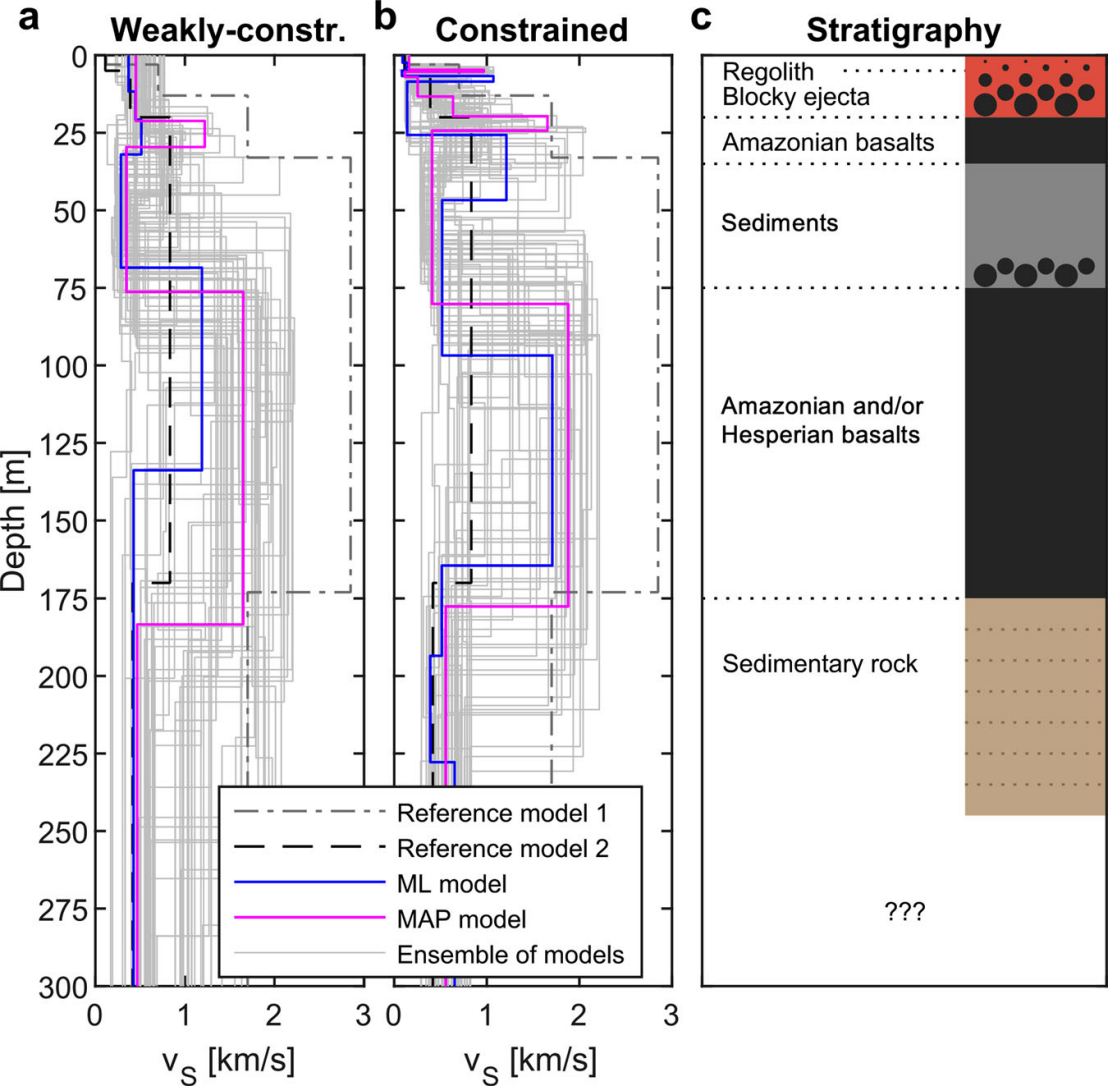
Interpretation of the seismic velocity models. **a**, **b** show *v*_S_ models for the weakly constrained (flat prior) and model-2 constrained inversion, respectively (see Figs. 5 and 6). The maximum likelihood (ML) and maximum a posteriori (MAP) models that both explain the observed ellipticity are displayed together with the two pre-landing subsurface models (gray dashed lines; reference models 1 and 2). Note that the low-velocity zone between around 30 and 75 m is a consistent feature found with both inversion approaches. Furthermore, note that the uppermost 20 m are not well resolved in both inversion runs. **c** Geological interpretation of the inferred models.

The low seismic velocities in the upper few meters of the surface are likely produced by an impact-fragmented regolith built up by cratering of basalts and eolian processes during the Amazonian after the formation of the Homestead hollow crater, where InSight is located, about 400−500 Myr ago^11,13,51^ (regolith layer in Fig. 7c). The regolith is dominated by sand-sized particles that are mostly unconsolidated with low densities, based on interpretations of thermal inertia and observations of soils (and few rocks), and thermal conductivity measurements around the lander^13,52,53^. Estimates of the thickness of this mostly sandy regolith layer are based on the source depth of observed ejecta. Fresh 30−60-m-diameter craters with non-rocky ejecta in the vicinity of InSight suggest a variable, but likely around 3-m-thick regolith layer near the lander^12,52^.

High-resolution images of scarps in similar terrain nearby indicate that this relatively sandy surface unit grades into coarse breccia and then jointed bedrock^51^. The uppermost meter of regolith is most likely finer-grained material than at deeper levels because small impacts generally break up the near-surface material more readily than at deeper levels where fewer large impacts penetrate. Within the topmost fine-grained sand layer, the seismic velocity increase is governed primarily by compaction^44^. At some depth, basaltic blocky ejecta with a higher seismic velocity (*v*_S_ of around 1800 m/s^44,54^) mixed with the fine-grained sand will lead to an increase of the bulk seismic velocity (blocky ejecta layer in Fig. 7c). The significant velocity increase at 1–2 m depth required by the compliance inversions^9^ reflect this change in regolith composition, or a significantly shallower regolith thickness at the landing site than suggested by orbital observations.

### Amazonian and Hesperian basaltic lava flow units

Beneath the lander, the top of the basaltic bedrock is estimated to be below 3 m depth. Geological mapping reveals volcanic vents and flow fronts that partially fill large craters^13^, mafic mineral spectra^46^, and the presence of wrinkle ridges, which have been interpreted as fault-propagation folds in weakly bonded, but strong layered materials such as basalt flows^55^. The thickness of the Amazonian (1.7 Ga) and Hesperian (3.6 Ga) basalt flows^51,56^ has been estimated from the lack of rocks in the ejecta of large (>2 km) fresh craters^13^ and from depth- and rim height−diameter relations of partially filled older and larger craters^46^. Near the lander, these estimates indicate that the Amazonian and Hesperian basalts are 160−180 m thick. These basalts are consistent with the high subsurface seismic velocities above about 175 m (likely *v*_S_ > 1800 m/s)^54^ and the thickness estimates from partially filled craters (Amazonian and Hesperian basalts in Fig. 7c). Beneath the basalts, phyllosilicate-bearing layered sedimentary rocks have been documented in the central peaks of large impact craters^46^. These physically weak sedimentary deposits are likely of Noachian age (>3.7 Ga) and are probably responsible for the lower seismic velocities below the Hesperian basalts at 175 m depth (sedimentary rock in Fig. 7c).

### Low-velocity sedimentary unit sandwiched between lava flows

The discussion above thus could readily explain the low seismic velocities in the top few meters (sandy, impact-fragmented regolith), the higher seismic velocities between 25 and 175 m (strong layered basalt flows), and the lower seismic velocities below 175 m (sedimentary deposits). The existence of the low-velocity zone spanning between around 30 and 75 m depth requires further explanation. The top of this low-velocity zone is less well resolved and is located at a depth somewhere between 25 and 40 m, whereas the bottom, located somewhere between 75 and 90 m depth, is a robust feature generally present through the whole ensemble of solutions and also different inversion settings.

The age of the basalt flows beneath the lander has been estimated from the size-frequency distribution of craters and cratering production functions. For craters with diameter above 2 km, the crater numbers indicate an Early Hesperian age (~3.6 Ga)^51^. However, for craters with diameter 200−700 m, the crater distribution suggests an Amazonian age (~1.7 Ga)^51,56^, indicating a younger resurfacing. As a result, there are ~2 Ga between the deposition of the Hesperian basalts and the younger resurfacing Amazonian basalts. To the south of the InSight lander, there are Noachian through Hesperian transition units^10^ that indicate active erosion and deposition of sedimentary materials near the dichotomy boundary of the southern Noachian highlands and the northern plains. To the east, Amazonian-Hesperian transition units include the Medusae Fossae Formation^10^, which is older than the Amazonian basalts. To the south, some of the Amazonian-Hesperian transition units^10^ are sedimentary deposits at least 10−30 m thick^46^ and alluvial activity has occurred further south in the Gale crater during the interval between the deposition of Hesperian and Amazonian basalts beneath InSight^57,58^. As a result, it is reasonable that the low-velocity zone spanning between 30 and 75 m depth could be a layer of sedimentary deposits sandwiched either between the Hesperian and Amazonian basalts (sediments in Fig. 7c) or somewhere within the Amazonian basalts.

## Discussion

Surface waves generated by sources like the interaction of wind with topography are ubiquitous on Earth, and are expected to be present on Mars. Regional-scale aeolian activity modeling around the Mars 2020 landing sites showed that strong winds are expected sol-around at topographic slopes^59^. Because the local topography around the InSight lander is relatively flat, the surface-wave source regions are likely located at regional distances around InSight, such as large craters and/or the topographic step of the dichotomy, though currently we cannot determine their exact locations.

As surface-wave trains propagate along the surface of a planet, the local subsurface structure on the scale of a few wavelengths around an observation point modifies their characteristics, leading to, for example, dispersion phenomena^60^. Low-velocity zones result in channel waves (trapped waves) and the build-up of amplitude for dispersed wave trains propagating as normal modes, generally termed Airy phase^60^. Airy phases are stationary phases associated with a minimum in the frequency-dependent group velocity that can propagate over considerable distances. For example, for continental travel paths, Airy phases at periods of 15−20 s are a prominent feature of terrestrial seismograms^61^. On local scales, Airy phases related to (low-velocity) coal seams are a well-studied phenomenon^62^.

We find that a low-velocity layer is required to explain the observed ellipticity curve. We interpret the prominent peak at 2.4 Hz observed in the vertical-component SEIS data as an Airy phase with associated amplitude build-up. The modeling of group and phase velocities for representative 1D velocity models displayed in Figs. 5 and 6 shows a clear group-velocity minimum at 2.4 Hz (see Supplementary Fig. 5).

This paper focuses on ambient vibrations, though the amplification of the vertical-component motion at 2.4 Hz has also been observed in broad-band and high-frequency marsquake recordings^3,4,63^. These observations of a consistent excitation of the 2.4 Hz peak during events can be readily explained by body-to-surface wave conversions close to the receiver at, for example, topographic features (e.g., craters) and/or shallow subsurface heterogeneities. Such body-to-surface wave conversions are commonly observed on Earth^64–66^.

## Methods

### Data preparation

The 2.4 Hz mode is a very stable feature in the SEIS dataset and is persistently visible on the recordings of Insight’s SEIS VBB (very broad-band) seismometer during quiet periods^17^. We investigated the variation of seismic background vibrations around and including the 2.4 Hz mode for the entire mission (see Fig. 1). Figure 2 illustrates the large variability of the noise recorded by SEIS during one sol. Windy time periods are not only characterized by elevated ambient noise levels but also prominent lander-related modes. In contrast, evening hours are usually very quiet with winds largely absent.

For the analysis reported here, we focused on a quiet 7-h long window of 100 samples-per-second VBB data recorded on the evening of Sol 422 (03/02/2020, 14:15 to 21:15 UTC, Sol 422 18:08–Sol 423 00:57 LMST; Fig. 2). We performed polarization analyses in the time-frequency domain^37,38^ of the entire data window. The polarization attributes (azimuth and tilt angles of the dominant motion^38^) displayed in Supplementary Fig. 2 reveal that the particle motion in this window and at 2.4 Hz is predominately elliptically polarized, with no preferred propagation azimuth, and a vertical to near-vertical orientation of the semimajor axis of the ellipse. Hence, the observed polarization indicates that elliptically polarized Rayleigh waves are the dominant component of the ambient seismic vibration at 2.4 Hz within our analysis window.

In this window, the lander-related modes were not excited. The H/V curves shown in Fig. 2b were calculated using the Geopsy software^33^. For each signal, the H/V curves for time windows of 120 s length were obtained according to

1 $$\frac{H}{V}\left(f\right)=\frac{\sqrt{{\left|E\left(f\right)\right|}^{2}+{\left|N\left(f\right)\right|}^{2}}}{\left|Z\left(f\right)\right|},$$

where *E*(*f*), *N*(*f*) and *Z*(*f*) are the spectra of the eastern, northern and vertical components, respectively. The final H/V curve for each of the three analysis windows is obtained as the geometric mean of the 120 s time windows.

We assume that we can retrieve Rayleigh wave ellipticity from the ambient vibration wavefield recorded by SEIS for the fundamental mode of Rayleigh waves using the RayDec method^28^. This method is based on the random decrement technique^67,68^ where the basic concept is as follows: (I) a narrow frequency filter is applied; (II) the zero-crossings from negative to positive amplitude values are searched for on the vertical-component signal; (III) time windows of a given length (corresponding to ten cycles at the given frequency in our case) are extracted on all three components, shifting the horizontal signals by a quarter-period of the selected frequency to compensate for the typical 90° phase shift of Rayleigh waves; (IV) the two horizontal components are projected in a direction maximizing the correlation with the vertical signal. Steps (III) and (IV) are repeated for each zero-crossing and the resulting vertical and horizontal signals are summed; (V) by calculating the square root of the ratio of the energies in the respective vertical and horizontal signals, the ellipticity at the given frequency is estimated; (VI) the processing is repeated for each frequency of interest. The RayDec processing suppresses other wave types than Rayleigh waves. Love waves are not present on the vertical component and are therefore supposed to be canceled out by the averaging process. Body waves do not show a phase shift between vertical and horizontal signals and should be suppressed as well. However, large contributions of other wave types on the horizontal components may lead to overestimated ellipticity values. The RayDec-derived ellipticity curve is shown in Fig. 4. The signal was cut in 10-min windows and each of them was analyzed independently. The resulting curve is the geometrical mean of these curves and the standard deviation is calculated accordingly.

In order to test the robustness of our Rayleigh wave ellipticity estimation, we compared the RayDec-derived ellipticity curve with other established techniques. Another method to estimate the Rayleigh wave ellipticity is the calculation of H/V using a time-frequency analysis (H/V TFA^69^). This approach performs a continuous wavelet transform of the three-component signals, finds the maxima on the vertical component and calculates the ratio between the 90° phase-shifted horizontal- and vertical-component signals at the identified times. In this way, transient Rayleigh waves are identified and their ellipticity is estimated. Supplementary Fig. 2 shows an overview of the analyzed Sol 422 signals using the classical H/V, RayDec and H/V TFA. In the Sol 422 data, the H/V curve shows the clear trough around 2.4 Hz and is relatively flat with values around 1 Hz at other frequencies between 1 and 8 Hz. At the trough frequency itself, the RayDec and H/V TFA approaches both yield very low ellipticity values of about 0.4. H/V TFA estimates even lower ellipticity values than RayDec in the other parts of the curve, but both are significantly lower than the classical H/V curve.

The extracted ellipticity values are low when compared with Earth sites. In theory, for a homogeneous half-space with a Poisson ratio of 0.25, the ellipticity of Rayleigh waves is about 0.68 at all frequencies^60^. As a boundary condition, the ellipticity of Rayleigh waves should approximate this value towards low frequencies. For realistic underground models where velocity increases with depth, the ellipticity value is frequency-dependent and shows a peak at the fundamental frequency of the site^23^. For sites with a strong velocity contrast, the peak frequency corresponds to a singularity in ellipticity, where the vertical-component signal vanishes and the ellipticity goes towards infinity^29^, followed by a trough at a higher frequency where the horizontal component vanishes and ellipticity goes towards zero. In the observed InSight data, we observe neither a singularity nor ellipticity values going towards zero at the trough. This provides a constraint on the velocity contrasts. In any case, ellipticity values below 1 are rarely observed over a wide frequency range on Earth and are indicative for low-velocity zone(s)^40,41^. For the inversion of the InSight data, we interpret the ellipticity values assuming that they reflect the characteristics of the Rayleigh wave fundamental mode in the frequency range from 1.5 to 8.0 Hz. The data errors estimated by the RayDec method were manually increased around 2.0 Hz and above 3.5 Hz (increase of 10% of the uncertainty multiplication factor in the logarithmic domain), to account for the higher harmonics of the 1-Hz electronic cross-talk noise^39^ and potential contamination with lander-related modes. These data errors work as an inverse weight in the inversion procedure, meaning a higher error results in a lower weight.

### Prior subsurface models to constrain the inversion

To constrain the generally ambiguous inversion of ellipticity curves, we took the near-surface (depths < 200 m) velocity models established before the landing^12,24,44,45^ and the ones based on first results^5^ as a starting point to establish suitable parameter space bounds of the inversion. A modified version of the near-surface model proposed by^24,45^ is summarized in Table 1 (model 1). The original model was condensed into a new model with fewer layers and the proposed low-velocity layer at 170 m depth was added. For this study, we updated model 1 in the following way to obtain the new conceptual model given in Table 2 (model 2): We expect to find a 3−6-m-thick low-velocity regolith layer at the surface that gradually transitions into a unit of coarse blocky ejecta. At a depth of a few tens of meters, heavily fractured basaltic rocks are expected, consisting of stacks of few to several tens of meters thick lava flows. Impact cratering is expected to be effective at cracking and breaking rocks at significant depth and we therefore assume relatively low seismic *v*_P_ velocities of around 1500 m/s for the basaltic units. Below the lava unit, at a depth of 150−200 m, a weak sedimentary unit has been suggested^13,46^. We expect rather low *v*_P_ velocities of around 750 m/s for these sedimentary rocks based on the observation that sedimentary rocks visited by rovers were observed to be very weak, even fissile. The *v*_S_ velocities were assigned keeping in mind the range of plausible values of Poisson’s ratio of such rock and soil materials. Still, neither of these conceptual reference models can explain the ellipticity trough at 2.4 Hz as shown by the forward modeling in Fig. 4.

### Bayesian inversion of the Rayleigh wave ellipticity curve

The inversion of the Rayleigh wave ellipticity curve to retrieve the underground 1D structure is a non-linear inverse problem characterized by significant inherent non-uniqueness as different models may fit the data equally well. Therefore, we perform the inversion of the ellipticity data in the Bayesian framework retrieving posterior probability on the presence of a particular wave velocity at depth. The applied inversion technique^32^ relies on a trans-dimensional formulation of the parameter space, where the number of layers is an unknown parameter itself^47,48^. The layered subsurface model is parameterized by a variable number of Voronoi cells with assigned values of *v*_S_, *v*_P_, and $\rho$. These assigned values, the positions of the Voronoi nuclei at depth, and the total number of layers are the sought parameters of the inverse problem. The number of layers is treated as self-adapting model complexity^49^ that is governed by the law of parsimony. The parameter space is explored by the Metropolis−Hastings algorithm^70^ with the implemented Parallel Tempering technique^71^. Furthermore, the multizonal formulation of the prior^32^ allows us to include depth-dependent prior expectations (i.e., minimal and maximal expected values in Tables 1 and 2) that constrain the inversion and restrict the ambiguous solution of this inverse problem. The result is an ensemble of solutions drawn from the posterior probability density function (posterior PDF) on the parameter space. Some representative velocity profiles may be selected (e.g., the maximum likelihood (ML) model providing the lowest data misfit or the maximum a posteriori (MAP) model estimate); nevertheless, they should be used only for illustration of the underground velocity pattern, in this case, as the ellipticity data do not carry information about absolute values of seismic velocities and depths.

### Inversion with flat prior

An inversion assuming strong prior expectations on the parameter space (prior PDF) may be misleading if the prior is not correct. Hence, we first performed an inversion of the ellipticity data in a parameter space with a flat prior PDF without any preference on layer depths or velocities (by setting the priors to a uniform distribution with relatively wide bounds). Such a weakly constrained inversion may still produce ambiguous and unrealistic models; however, it provides valuable information about the velocity profile pattern without an influence of the prior PDF. Besides, the implemented Parallel Tempering technique^71^ reduces the dependence on an initial (i.e., starting) model by using a large amount of parallel Markov chains with random and independent initial models. The result of such a weakly constrained inversion is shown in Fig. 5. The ensemble of solutions consists of more than 1,250,000 models that fit the ellipticity data well. The inversion result indicates that: (I) there are underground structures explaining the ellipticity data; (II) seismic velocities of the expected underground model in Table 2 (black dashed line) are reasonably within the range of likely values of the inferred posterior PDF (this does not apply to the model in Table 1); (III) a low-velocity zone at the depth of roughly 30−75 m is likely underlain by a compact thick high-velocity zone; (IV) the bottom of the compact high-velocity zone at a depth of around 170 m seems to be in agreement with the expectations from Table 2; (V) there might be a thin high-velocity zone located above the low-velocity zone; however, its properties are uncertain (cannot be uniquely resolved from data); VI) the histogram in Fig. 5e shows that at least five layers (four layers and half-space) are necessary to fit the data. Less than five layers do not fit the data sufficiently, while more layers do not provide significantly better data fitting.

### Multizonal constrained inversion

The inversion result displayed in Fig. 5 implies that the use of model 2 (Table 2) as the prior may not introduce a strong divergent effect in the inversion results. Hence as a next step, we performed an inversion of ellipticity data in a parameter space constrained by the bounds summarized in Table 2 (using a multizonal prior and allowing for low-velocity zones). The parameter space is constrained by means of the depth-dependent prior PDF having four zones with thicknesses and velocity bounds as presented in Table 2. The results of such a constrained inversion are shown in Fig. 6, where the ensemble of solutions consists of more than 1,350,000 models. The main features remain as observed for the weakly constrained inversion, which we interpret as a good sign regarding a possible effect on the posterior PDF. Additionally, a thin high-velocity layer is likely above the low-velocity zone even in this inversion with constrained seismic velocities. However, its thickness and depth remain very ambiguous and unclear. To conclude the inversion tests, the ellipticity trough at 2.4 Hz can be explained by at least one low-velocity zone within the expected thick volcanic layer (the basalt layer in Table 2). Such a low-velocity zone divides the volcanic layer into the upper (ambiguous) high-velocity layer, low-velocity zone, and bottom compact high-velocity zone (see inferred models in Fig. 7). Nevertheless, the ellipticity data reflect rather the velocity contrasts in depth than absolute values of velocities; hence, we can interpret only the velocity pattern.

### Additional supporting inversion tests

We have performed several Bayesian inversion tests with various settings to reveal the robustness of the inferred features. As examples, Supplementary Figs. 3 and 4 show additional inversion tests with a fixed number of layers (i.e., a standard non-trans-dimensional Bayesian inversion with a flat prior). In these two tests, the seismic velocity structure is without an influence of the prior, the initial models are random and independent, and the number of layers is fixed to five and six layers, respectively. These tests support the seismic velocity pattern as described above, and show that models with five layers (four layers above a half-space) are most suited to explain the observed ellipticity curve.

## Supplementary information

Supplementary Information

## Data Availability

The seismic waveform data that support the findings of this study are available from NASA PDS (National Aeronautics and Space Administration Planetary Data System, https://pds.nasa.gov/; InSight Mars SEIS Data Service, 2019; 10.18715/SEIS.INSIGHT.XB_2016). The Rayleigh wave ellipticity as well as supporting H/V data displayed in Supplementary Fig. 2 that form the basis for the presented study are available with the paper as supplementary files (MS Excel sheets).

## Code availability

The RayDec code used for data preparation (extraction of Rayleigh wave ellipticity values as a function of frequency) is available at https://github.com/ManuelHobiger/RayDec (10.5281/zenodo.5534777). The Geopsy open-source software^33^ (http://www.geopsy.org/) was used for all forward computations. The Rayleigh wave ellipticity curves were inverted using the software package MTI (ver. Desert) utilizing the Multizonal Trans-dimensional Bayesian Inversion developed at SED, ETH Zurich, available upon request. MTI uses some routines from the Parametric Slip Inversion^49^ available under the GNU General Public License, and the computer package Parallel Tempering (Inversion Laboratory, ilab), made available with support from the Inversion Laboratory (ilab, http://www.iearth.edu.au/), which is a program for construction and distribution of data inference software in the geosciences supported by AuScope Ltd, a nonprofit organization for Earth Science infrastructure funded by the Australian Federal Government. Data processing was done using ObsPy^72^, NumPy^73^ and SciPy^74^, figures were created using matplotlib^75^ and MATLAB^76^.
