# Supplementary material for: The shallow structure of Mars at the InSight landing site from inversion of ambient vibrations
Source: Nat Commun. 2021 Nov 23;12:6756. doi: 10.1038/s41467-021-26957-7 (PMC8611082; doi:10.1038/s41467-021-26957-7)
Supplement: Supplementary file 1 — Supplementary Information [file 41467_2021_26957_MOESM1_ESM.docx]

**SUPPLEMENTARY FIGURES**

**The shallow structure of Mars at the Insight landing site
from inversion of ambient vibrations**

M. Hobiger^1,2^, M. Hallo^1^, C. Schmelzbach^3*^, S. C. Stähler^3^,

D. Fäh^1^, D. Giardini^3^, M. Golombek^4^,

J. Clinton^3^, N. Dahmen^3^, G. Zenhäusern^3^, B. Knapmeyer-Endrun^5^, S. Carrasco^5^,
C. Charalambous^6^, K. Hurst^4^, S. Kedar^4^, W. B. Banerdt^4^

6 October 2021

*^1^Swiss Seismological Service (SED), ETH Zurich, Switzerland; ^2^now at: Federal Institute for Geosciences and Natural Resources (BGR), Hanover, Germany; ^3^Institute of Geophysics, ETH Zurich, Switzerland; ^4^Jet Propulsion Laboratory, California Institute of Technology, Pasadena, CA 91109, United States of America; ^5^Bensberg Observatory, University of Cologne, Bergisch Gladbach, Germany; ^6^Imperial College, London, Department of Electrical and Electronic Engineering, United Kingdom. Correspondence should be addressed to C.S. (email: cedric.schmelzbach@erdw.ethz.ch)*


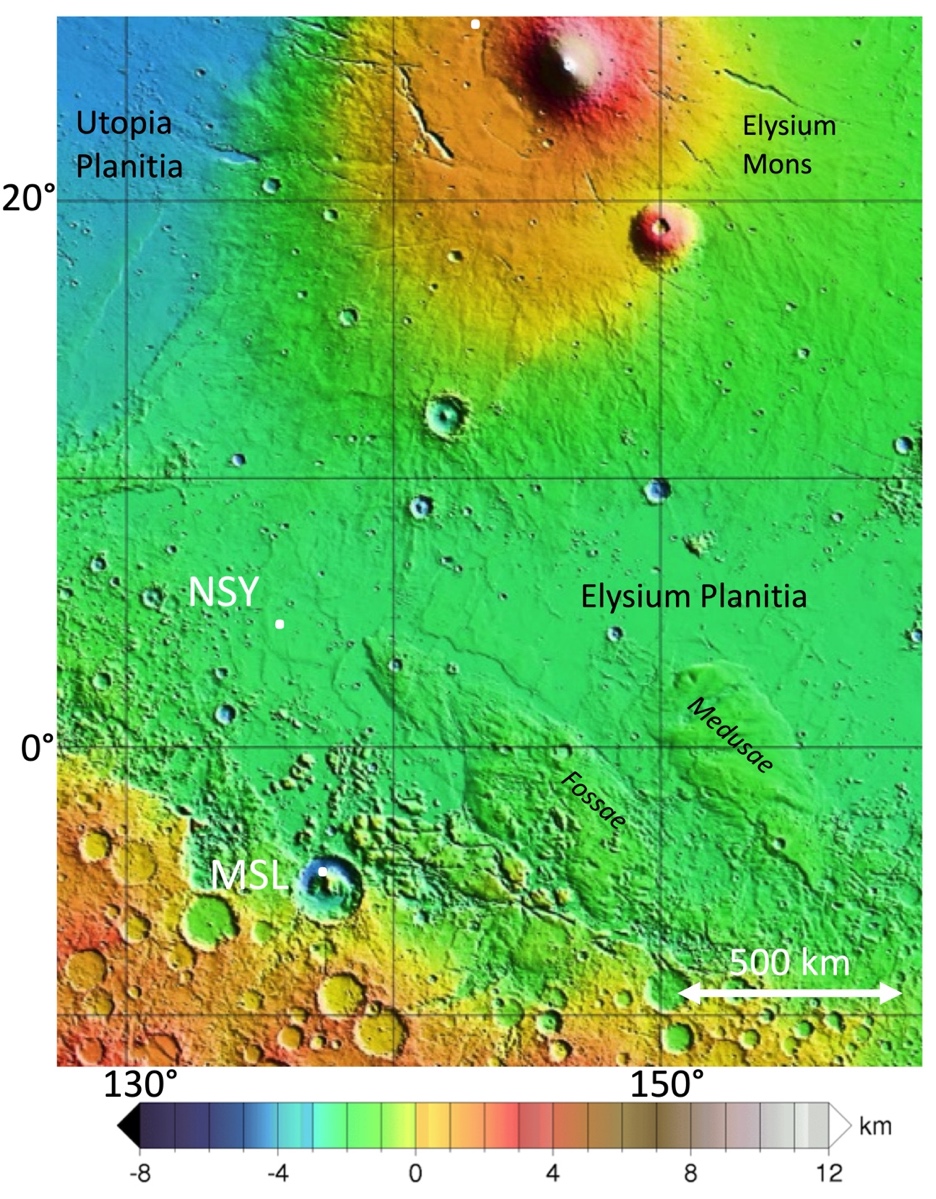


**Supplementary Figure 1.** Regional setting of the InSight landing site in western Elysium Planitia according to the Mars Orbital Laser Altimeter (MOLA) topographic map with elevations with respect to the areoid. The lander is on ridged plains north of the dichotomy boundary between the Noachian heavily cratered terrain to the south and the younger northern plains. The ridged plains are interpreted to be Hesperian volcanic basalt flows that were resurfaced in the Early Amazonian. Younger volcanics are associated with Elysium Mons to the north and from central Elysium Planitia to the east. Note the rough topography of the transition zone and the >3 km elevation offset of the dichotomy. Erosion resulted in sedimentary deposits in the transition zone in the Noachian and Hesperian, including the Medusae Fossae Formation. NSY: NASA InSight; MSL: NASA Mars Science Laboratory (Curiosity).


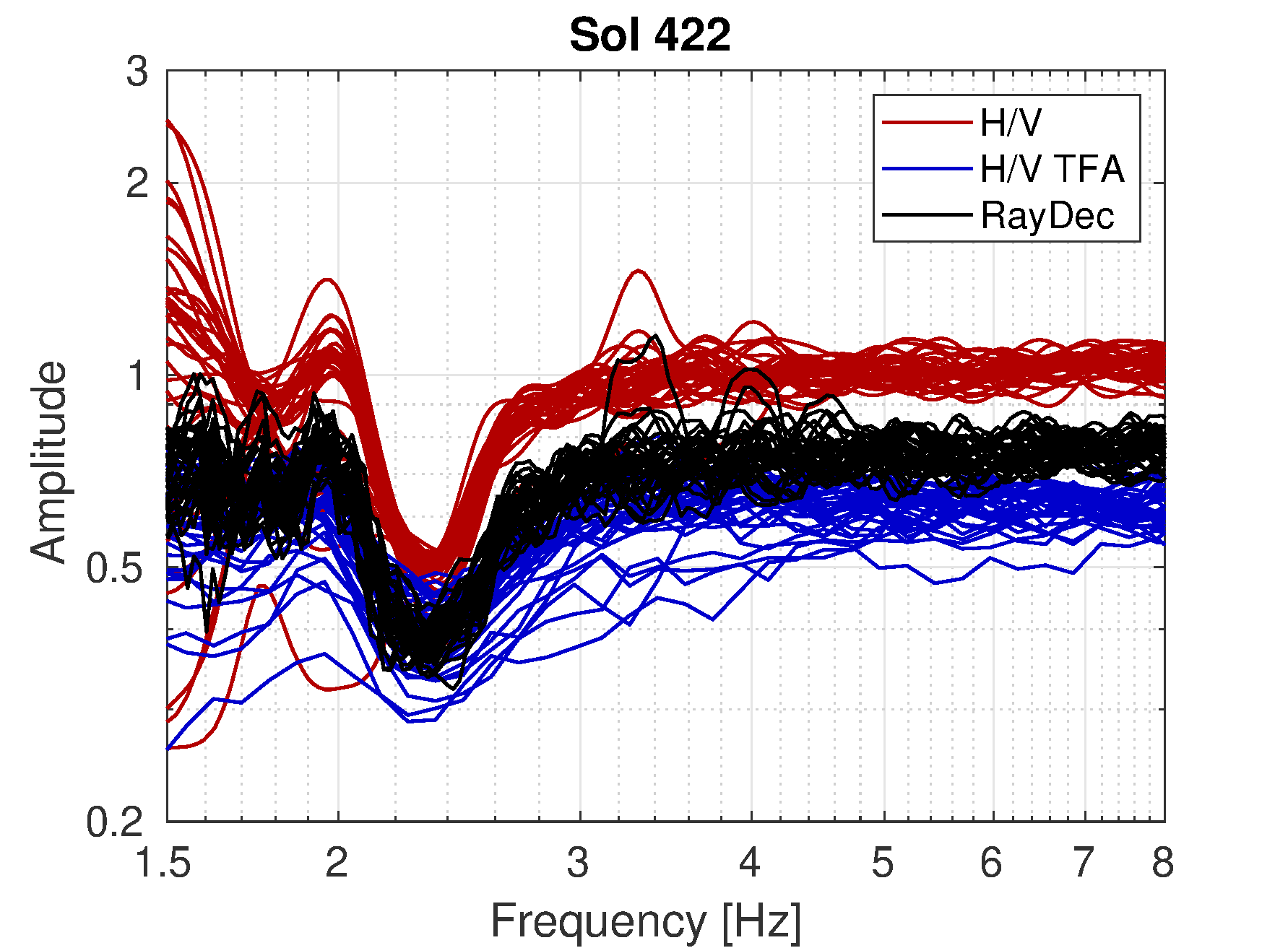


**Supplementary Figure 2**. Comparison of different approaches to estimate the H/V ratio and Rayleigh wave ellipticity: The classical H/V approach (Nakamura, 1989), H/V using the time-frequency analysis (Fäh et al., 2009) and RayDec (Single-station determination of Rayleigh wave ellipticity by using the random decrement technique; Hobiger et al., 2009).


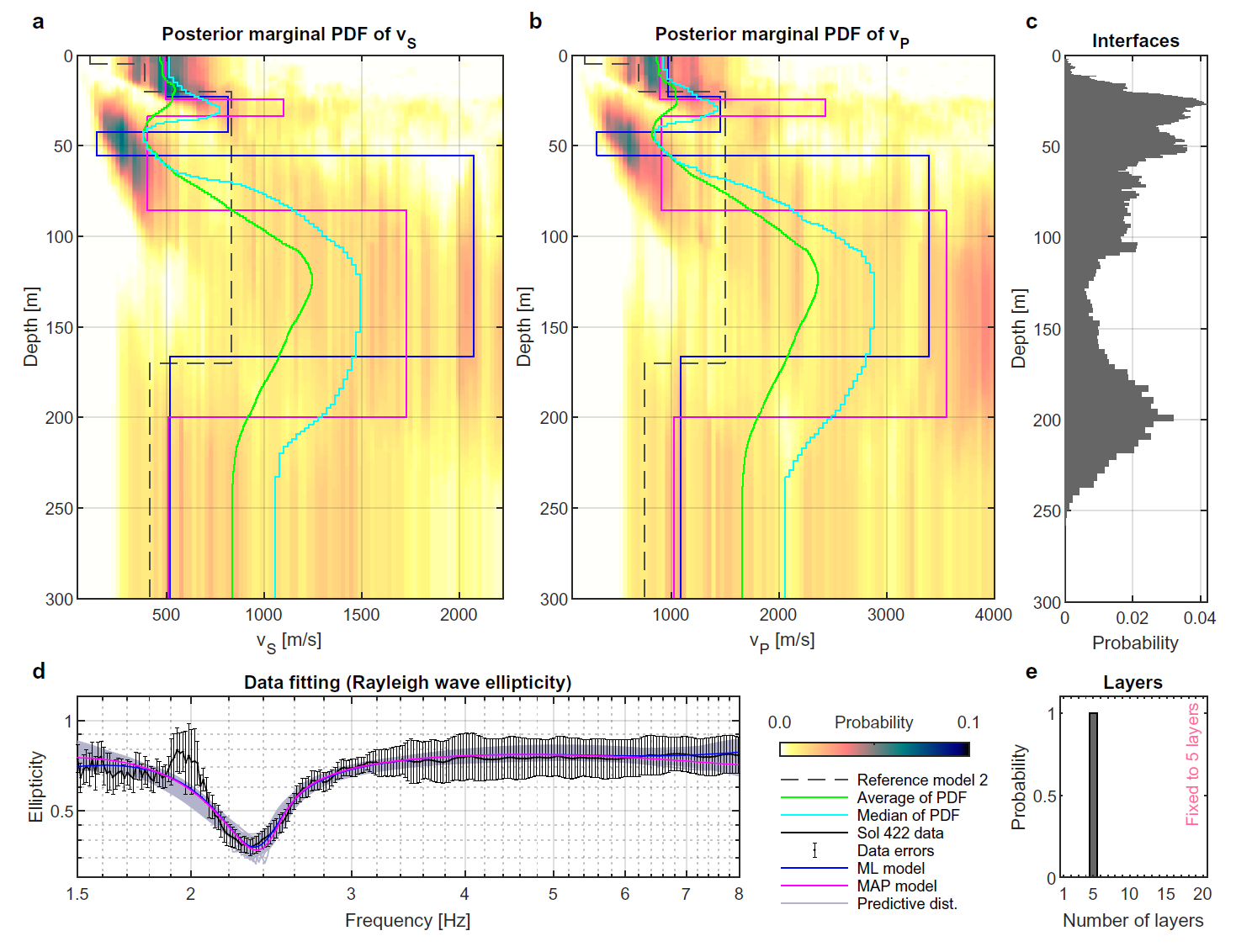


**Supplementary Figure 3.** Result of the inversion with constrained number of layers (5 layers with varying thicknesses, using flat prior probability density function (PDF) of seismic velocities). (a) and (b) show the posterior marginal PDFs of *v_s_* and *v_p_*, respectively. (c) Histogram of the occurrence of layer interfaces. (d) Extracted (black line) and modelled ellipticity curves (ML: maximum likelihood; MAP: maximum a posteriori). Vertical bars indicate the data error. (e) Posterior histogram of the number of layers.


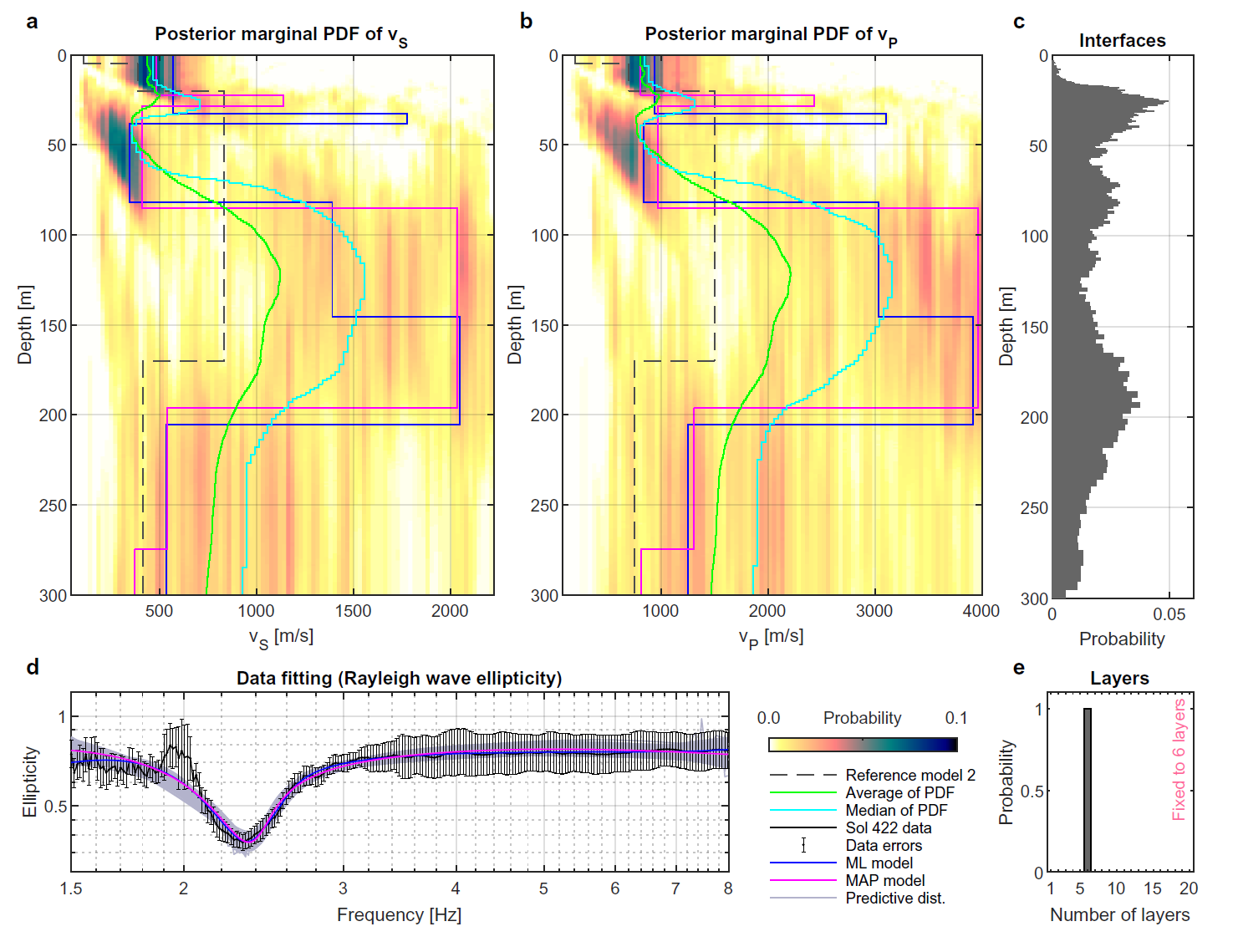


**Supplementary Figure 4.** Result of the inversion with constrained number of layers (6 layers with varying thicknesses, using flat prior probability density function (PDF) of seismic velocities). (a) and (b) show the posterior marginal PDFs of *v_s_* and *v_p_*, respectively. (c) Histogram of the occurrence of layer interfaces. (d) Extracted (black line) and modelled ellipticity curves (ML: maximum likelihood; MAP: maximum a posteriori). Vertical bars indicate the data error. (e) Posterior histogram of the number of layers.


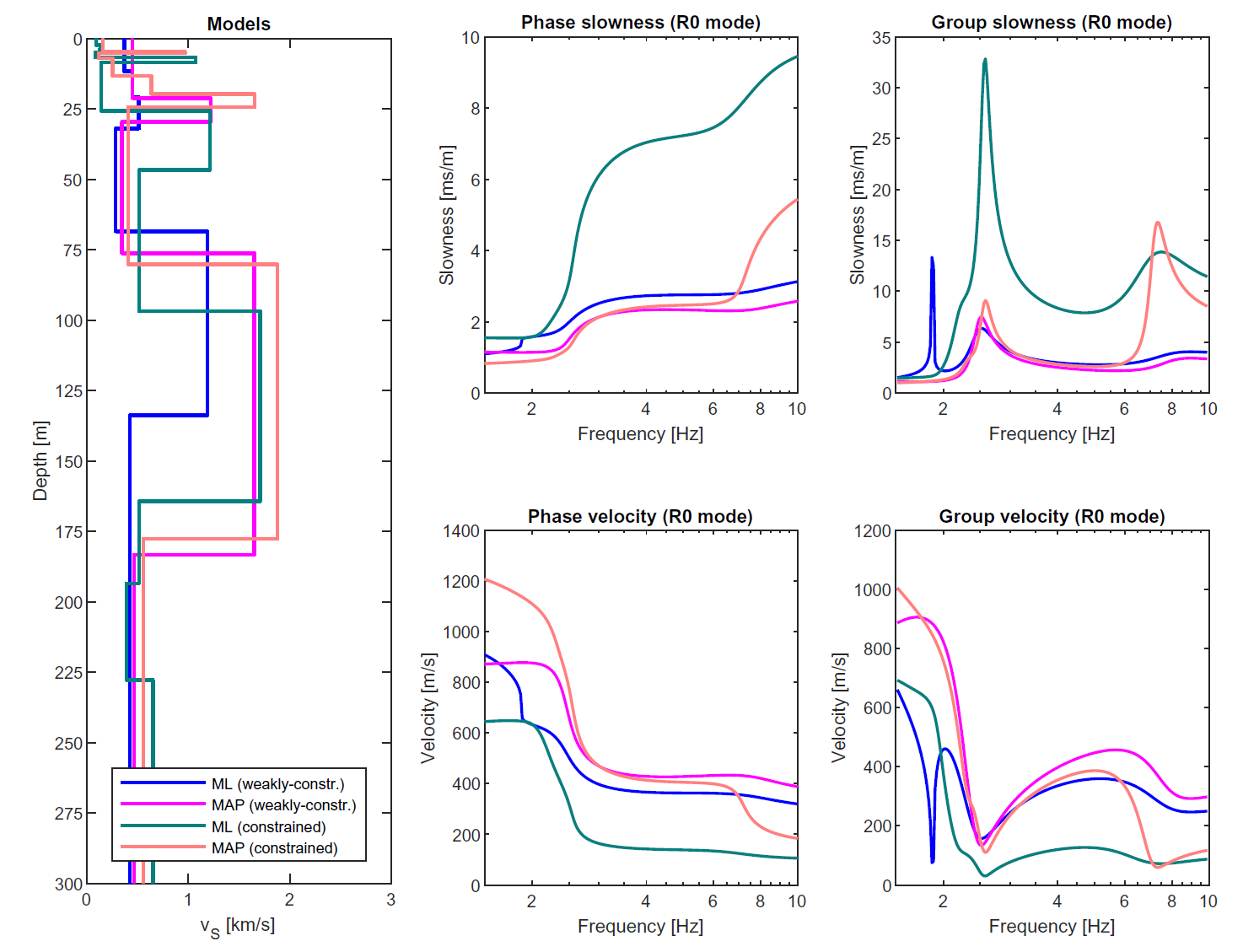


**Supplementary Figure 5**. Predicted fundamental-mode Rayleigh wave (R0 mode) dispersion curves computed for the different inferred models shown (left panel; ML: maximum likelihood; MAP: maximum a posteriori). The predicted curves are displayed in phase and group slowness (top right), and phase and group velocity (bottom right). Note the prominent group-velocity minimum at 2.4 Hz indicative of an Airy phase.
